# Depression symptoms, communication and cooperation skills, and friendship: longitudinal associations in young Norwegian children

**DOI:** 10.3389/frcha.2024.1328527

**Published:** 2024-08-22

**Authors:** Amanda Krygsman, Tracy Vaillancourt, Harald Janson, Thormod Idsoe, Ane Nærde

**Affiliations:** ^1^ Counselling Psychology, Faculty of Education, University of Ottawa, Ottawa, ON, Canada; ^2^ School of Psychology, Faculty of Social Sciences, University of Ottawa, Ottawa, ON, Canada; ^3^ Norwegian Center for Child Behavioral Development, Oslo, Norway; ^4^ Department of Special Needs Education, Faculty of Educational Sciences, University of Oslo, Oslo, Norway

**Keywords:** depression symptoms, communication, cooperation, best friend, early childhood

## Abstract

**Introduction:**

Symptoms of depression in early childhood have been linked to interpersonal difficulties, whereas friendships serve a protective function.

**Methods:**

In the present study, we examined depression symptoms in preschool age (4 years) in relation to social skills (communication and cooperation), and friendships into early school age (Grades 1 and 2) in a large subsample (*n *= 943) of Norwegian children.

**Results:**

The results indicated that preschool depression symptoms negatively predicted Grade 1 communication skills, which in turn predicted Grade 2 depression symptoms. This pathway suggests that communication skills may be a maintenance factor for depression symptoms in young children. In addition, preschool depression symptoms predicted lower Grade 1 cooperation skills, which in turn predicted lower Grade 2 communication skills, suggesting that preschool depression symptoms may begin a cascade of social skill problems that affect cooperation and communication skills into early school years. Best friendships were negatively related to depression symptoms in preschool and Grade 2.

**Discussion:**

Given that preschool depression symptoms impact the development of social skills and friendships, it is important to attend to depression symptoms in early childhood.

## 1 Introduction

There is growing evidence of symptom-driven pathways of internalizing symptoms predicting social and emotional difficulties, such as problems with peers and further internalizing symptoms in early and late childhood and adolescence (1–3). Depression symptoms can occur as early as preschool age with young children showing similar symptom patterns (adjusted for age-appropriate expressions) as older children and adults (2, 4–6). Depression symptoms also show continuity into the school years (7, 8). Interpersonal difficulties (i.e., social problems) are strongly linked to depression symptoms at all ages (9), while good interpersonal relations like friendships are protective (10). In the present study, we were interested in the longitudinal relations between depression symptoms in preschoolers and the development of social skills (i.e., cooperation and communication) and friendship across Grades 1 and 2.

Interpersonal models of depression suggest that relational processes relate to the development and/or maintenance of depression (11–13). For example, children with depression symptoms may engage in interpersonal behavior that others find aversive, leading to rejection by peers and/or adults, which in turn relate to subsequent depression symptoms (11–13). Depressed individuals may exhibit paralinguistic types of behavior that can interfere with attempts to communicate with others. In particular, they tend to have slower speech, say less, be quieter, and take more time to respond to the speech of others (9). Others rate the speech of those experiencing depression as less clear, and more difficult to hear and to understand than non-depressed individuals (9). These features may negatively influence communication with and responses of others. The actual content of speech also tends to be more negative in depressed individuals and negative self-disclosures more common, which have been linked with rejection (9, 11). Although much of the experimental evidence regarding paralinguistic behavior has been established in adults, depression symptoms linked to these deficits have also been shown to occur in preschool children (e.g., anhedonia, psychomotor agitation, irritability, cognitive impairment) (4).

Early school-aged children experiencing depression symptoms have also been found to be less social, spend more time alone, are more rejected by peers, and display more hostility (14, 15). Researchers have found that friendship is protective for those at genetic risk for depression symptoms, particularly for girls when friendships are reciprocated (10). This suggests that early depression symptoms may be a risk for later depression symptoms and that peer problems (7) and social skill development (9) may maintain symptoms over time.

Earlier theories of depression typically linked deficits in social skills to the development of depression symptoms (e.g., 16). More recent theories, however, note that what appears as a deficit may in fact be a lack of expression of a particular skill during a depressed state rather than an enduring deficit that is also present when depression symptoms are low (9). Interpersonal theories of depression posit that social-behavioral deficits and relationship problems are predicted by depression symptoms in late childhood and adolescence (3). This pathway has also been found in preschool-aged children, although less commonly examined (e.g., 2). The ability of a preschool child to respond in a socially competent manner can be compromised when high levels of negative emotions occur in their social interactions (17). High levels of negative emotions are common for preschoolers with depression symptoms (5). Emotional competence is concurrently associated with social competence in preschool age and longitudinally associated with social competence in kindergarten (18).

Deficits in social skills can be related to acquisition of the skill (acquisition deficits) or the ability to perform a skill (performance deficits), whereby the child knows how to perform the skill but chooses not to do so in certain settings or circumstances (19). Many social skills deficits are now understood as motivational problems rather than a lack of knowledge to execute the skill and, consequently, that performance deficits can arise when the skill has not been adequately reinforced (19). Two social skills that are particularly influenced by intervention programs are cooperation and communication (20), which are typically learned, practiced, and established through forming and maintaining friendships, and particularly having a best friend.

Depression symptoms already occurring in preschool may interfere with the acquisition and performance of social skills such as communication and cooperation. Preschoolers’ depression symptoms can also influence their school-aged cooperation and communication skills. Conversely, poorly developed social skills may impact depression symptoms. Depression symptoms may also influence the ability to form friendships directly or have friends indirectly through poorly developed cooperation and communication skills. Having friends may further impact a subsequent improvement of cooperation and communication skills. Friendships are dyadic, reciprocal, and voluntary, and friends are typically the relationship that is most enjoyable and that matters most to children (21–23). By the age of 4–5 years, three-quarters of children have a close friend and can reliably identify a best friend (24).

Best friends are normally expected to spend more time and share more resources (e.g., support, intimacy) than other friends (22). Having a best friend in preschool thus allows for more positive social experiences and is a meaningful relationship at this age (25). Friendships in early childhood tend to center on common activities and concrete reciprocity (22). Children with higher depression symptoms generally have peer interactions of lower quality, including less cooperation (15), and their play partners often conclude that the depressed child does not enjoy playing with them (26). Negative content in conversation and unsolicited negative feelings also tend to occur more frequently in close relationships of those with depression, which are often expressed at times that are socially inappropriate, placing them at risk of not maintaining close friendships over time (9, 11, 27).

Preschoolers with better communication and cooperation skills are generally more accepted by their peers, whereas those who are withdrawn tend to be less accepted by the peer group (28), resulting in fewer opportunities to practice the skills needed to maintain friendships, including cooperation and communication (29). In fact, communication skills are core skills that underpin the development of friendships in early childhood and cooperation is a fundamental skill that helps establish and maintain friendships (30).

Despite the established links between depression and social competencies (and deficits) in childhood, there is a paucity of research examining the direction of association addressing early social competencies (e.g., having a best friend, cooperation, communication) and depression symptoms. Accordingly, we explored associations between teacher-reported child depression symptoms and friendship in early childhood and school-age social skills and friendship. Specifically, we used a cross-lagged panel model (CLPM) to examine how depression symptoms were associated with cooperation and communication skills, along with having a best friend across early childhood in a large population-based sample of Norwegian children.

Parent internalizing (i.e., anxiety and depression) symptoms, early socioeconomic risk, child gender, as well as hours of daycare attendance were explored as covariates given that parental symptoms of anxiety and depression have been associated with children's depression symptoms (31), that gender differences in cooperation skills have been found for young children (32), and because the protective factor of friendship is suggested to have more impact on depression symptoms for girls (10). To account for these associations, we included the covariates as part of our model.

## 2 Materials and methods

### 2.1 Participants

Data were selected from the Behavior Outlook Norwegian Developmental Study (BONDS), a longitudinal study that began when children were aged 6 months (*N* = 1,159) (33, 34). Data used in the current paper were collected from parents, daycare teachers, and schoolteachers at the child’s age of 6 months, 12 months, 4 years [M_age _= 50.28 months (SD_age _= 1.44)], in Grade 1 [M_age _= 77.56 months (SD_age _= 3.34)], and in Grade 2 [M_age _= 98.53 months (SD_age _= 3.29)]. Most children were in daycare at the age of 4 years (97.8%) and 5 years (98.4%). Most daycare teachers reporting on children at 4 years had the position of educational leader (80.5%) and had a preschool teacher education (81.8%), whereas schoolteachers reporting at Grades 1 and 2 had teacher training (i.e., post-secondary education). In Norway, children start school (Grade 1) in August, the calendar year they turn 6 years of age. Before formal schooling and starting from age 1, Norwegian children have access to universal and quality regulated kindergarten (i.e., preschool in North America), which is highly subsidized. Today, most toddlers and preschoolers in Norway attend kindergarten, including more than 97% of all children aged 3–5 years, and 85% of children aged 1–2 years (35). Children can be placed in kindergarten from the age of 1 until the summer of the calendar year they reach 6 years of age.

### 2.2 Procedures

Trained research assistants interviewed mothers and fathers in local offices or in their homes, if preferred. Although both parents were invited to participate in the first interview when their child was aged 6 months and at 48 months, due to limited resources and to reduce dropout, fathers were primarily targeted when their child was aged 12 months and in Grade 1 and mothers when their child was in Grade 2 [see Nærde et al. (34) for more details]. The daycare teachers who knew the target child the best filled out questionnaires at the daycare center when child participants were aged 4 years and the child's main teacher filled out questionnaires around December of Grades 1 and 2.

### 2.3 Measures

#### 2.3.1 Depression symptoms

The ASEBA Teacher Report Form (TRF) (36) withdrawal/depression subscale (age 4 years: C-TRF 1.5–5; 10 items; e.g., “Shows little interest in things around him/her”; Grade 1 and 2: C-TRF 6–12; 8 items; e.g., “There is very little that he/she enjoys”) was used to measure depression symptoms in young children. Items were rated along a 3-point scale (0 = not true; 1 = somewhat or sometimes true; 2 = very true or often true). The items were averaged to create a composite score of depression symptoms. Cronbach's alpha was 0.79 [95% confidence interval (CI) 0.77–0.82] at age 4, 0.75 (95% CI 0.73–0.78) in Grade 1, and 0.74 (95% CI 0.71–0.76) in Grade 2. Cronbach's alpha was in the range of 0.7–0.9 (37) and the lower bound of the 95% confidence interval was greater than 0.7 (38), which provide support that the reliability of the scales were likely acceptable. A validated Norwegian version of the TRF was administered to participants (39).

#### 2.3.2 Cooperation and communication skills

Cooperation and communication in Grades 1 and 2 were measured using the teacher version of the social skills scale from the Social Skills Improvement System Rating Scales (SSIS-RS) (40), encompassing two of the seven included subscales. Items were rated along a 4-point scale (0 = never; 1 = seldom; 2 = often; 3 = almost always) and items were summed to form a composite score. Cronbach's alpha for cooperation (six items; e.g., “Follows classroom rules”) was 0.88 (95% CI 0.86–0.89) in Grade 1 and 0.89 (95% CI 0.87–0.90) in Grade 2, and for communication (seven items; e.g., “Takes turns in conversations”) it was 0.80 (95% CI 0.78–0.82) in Grade 1 and 0.83 (95% CI 0.81–0.84) in Grade 2. The Cronbach's alpha was in the range of 0.7–0.9 and the lower bound was greater than 0.7, suggesting that the values were likely acceptable. We used a Norwegian translation of the SSIS-RS by researchers at the Norwegian Center for Child Behavioral Development using a backtranslation procedure.

#### 2.3.3 Best friend

Teachers reported on participating children's best friendship by answering the question: “Does this child have a special friend or best friend in kindergarten?” (age 4) and “Does this student have a special friend or best friend in the class?” (Grades 1 and 2) using response options of 1 = yes and 0 = no. These items were administered in their original Norwegian form.

#### 2.3.4 Covariates

##### 2.3.4.1 Parent internalizing symptoms

The 12-item short version (41) of The Hopkins Symptom Checklist (SCL) (42) was used to measure parental anxiety (e.g., Feeling fearful) and depression symptoms (e.g., Feeling of worthlessness) at age 4 for both fathers and mothers. Items were answered along a 4-point scale (1 = not at all bothered; 2 = bothered a little bit; 3 = quite a bit bothered; 4 = very much bothered) and averaged to form a composite score. Cronbach's alpha was in acceptable ranges of 0.88 (95% CI 0.87–0.89) for fathers and 0.90 (95% CI 0.88–0.91) for mothers. The SCL was presented to participants in Norwegian and was a validated Norwegian version (41).

##### 2.3.4.2 Early socioeconomic risk and hours in daycare

Early socioeconomic risk was measured using a cumulative risk score calculated from socioeconomic status items from the ages 6 and 12 months on parent education and occupation, financial difficulty, housing conditions, and lone parent status (43). Specifically, low parental education [response options included: “9-year elementary school or shorter,” “1–2 years of high school,” “Higher vocational,” “3 years of high school, upper secondary school,” “College, university up to 4 years (Bachelor, Nurse, Teacher, Engineer),” or “University, college, more than 4 years (Master's degree)”] where at least one parent with 1–2 years of high school or less was classified as low parental education, at least one parent unemployed and/or on welfare or staying at home (response options included “occupationally active,” “student or school pupil,” “parental leave,” “stay at home,” “unemployed, looking for work, on measures,” “social security, under rehabilitation”), having endorsed enduring financial hardship over the previous year [item: “Now there are some questions related to various strains” with a response option: “Finances (payment of rent, loans, obligations, and the like)”], endorsement of two or more of the following three housing conditions were classified as high risk: not owning a home, or a home with fewer than two bedrooms, or dissatisfaction with the home (item: “How many bedrooms and living rooms does the home have? (Living room of 6 m^2^ or more, apart from kitchen, bathroom, hallway, laundry room)” where participants answered the number of rooms and “How satisfied are you with the home you live in?” with response options of “very satisfied,” “satisfied,” “dissatisfied,” “very dissatisfied”), and the parents not living together or the mother being single, widowed, or separated from the father (response options included: “married or registered partner living together,” “cohabiting,” “single/divorced/separated/widow/widower”). A risk score was calculated for each item (i.e., high risk = 1 or low risk = 0) and summed to form a composite score in the range of 0–5. At age 4, parents were also asked “How many hours per week does the child spend out of the home (includes time in a day care center, and time with a babysitter).”

### 2.4 Analytic plan

Participants with at least one data point over time on each of depression symptoms, cooperation skills, communication skills, and having a best friend were selected for the analytic sample (*n* = 943; 81.4% of the original sample). A CLPM was performed in Mplus version 8.0 (44) with a weighted least squares (WLSMV) estimator due to the best friend items being dichotomous and endogenous. Models were evaluated using a comparative fit index (*CFI*) of 0.95 or above as adequate and root mean square error of estimation (*RMSEA*) of less than 0.08 as adequate (45, 46). The chi-square test was used as a fit indicator while considering the sensitivity to large samples (47). The WLSMV estimator requires the chi-square test to be calculated using the DIFFTEST option in Mplus to compare nested models only (44). Using this estimator, the least restrictive model is estimated first and then any model restrictions are compared to the overall model.

We began with an overall model (Model 1) including covariance paths within time point and stability paths across the same construct over time, as well as 1-year cross-lagged paths among depression symptoms, cooperation skills, communication skills, and having a best friend and covariates (i.e., early socioeconomic risk from birth to 1 year of age, parental internalizing symptoms at age 4, and hours in childcare at age 4) were included. In this model, gender, early socioeconomic risk, and parental internalizing symptoms were predictors of each of the variables in our model, whereas hours in childcare at age 4 was a predictor of the included variables at age 4 (i.e., depression symptoms and having a best friend). Covariates were allowed to be correlated. Although the Akaike information criterion (AIC) is typically an indicator of model fit related to non-nested models, this indicator was not calculated when the WLSMV estimator was used. Therefore, non-nested models with and without covariates could not be compared using model fit indices available and covariates were added to the overall model.

In subsequent models, any paths that were similar across time (e.g., cooperation with communication in Grades 1 and 2) were constrained to be equal and compared to the overall model. Any constraints that did not have a statistically significant change in fit were retained in the model and any constraints that resulted in a statistically significant change in chi-square were interpreted as degrading model fit and therefore were allowed to remain freely estimated to obtain the most parsimonious model. Indirect effects (statistically significant paths across three time points) were examined using the MODEL INDIRECT command in MPlus and 95% bootstrapped (5000) confidence intervals that did not include 0 interpreted as statistically significant.

Little's missing completely at random (MCAR) test was conducted to describe any pattern(s) of missing data. When the MCAR test was not statistically significant, MCAR was assumed, and when it was statistically significant, then the underlying *t*-tests comparing missingness among variables were examined to delineate any missing data patterns that may impact results and interpretation.

Covariates were compared using *t*-tests for continuous variables and chi-square tests for dichotomous variables based on those included or excluded from the analytic sample. Gender differences were explored using a multi-group model and were treated as a covariate upon convergence issues.

## 3 Results

### 3.1 Missing data

Most values of skewness and kurtosis were under the recommended ranges (i.e., under 3 for skewness and under 10 for kurtosis) except for child depression symptoms in Grade 1, which was slightly above 10.81 and deemed to be acceptable (47). Little's MCAR test was statistically significant [*χ*^2^(101) = 128.104, *p* = 0.032]; however, none of the underlying *t*-tests were statistically significant. The participating parents with data included in the analytic sample (*n* = 943) were compared to those who were not included (*n* = 216). Parents experiencing greater early socioeconomic risk were less likely to be included in the analytic sample [*t*(203) = 3.258, *p *< 0.001; 30.3% one or more risk factors in the analytic subsample vs. 40.3% in those not included]. There were no differences in parental internalizing symptoms scores [*t*(1,058)=−0.255, *p *= 0.399], hours in daycare [*t*(156)=−1.55, *p *= 0.062], or in the gender of the child [*χ*^2^(1) = 0.015, *p *= 0.91] between those included and those not included in the analytic sample. There was no evidence of problematic bias related to missing data patterns and thus we proceeded with our analytic plan.

### 3.2 Descriptive statistics

The means, standard deviations, and correlations for the study variables are provided in Table 1. Depression symptoms were associated with not having a best friend within each time point (age 4: *r *= −0.12, *p *< 0.01; Grade 1: *r *= −0.08, *p *< 0.05; Grade 2: *r *= −0.11, *p *< 0.01). Having a best friend was associated with better communication and cooperation skills within time (Grade 1: communication: *r *= 0.13, *p *< 0.01; cooperation: *r *= 0.11, *p *< 0.01; Grade 2: communication: *r *= 0.07, *p *< 0.05; cooperation: *r *= 0.12, *p *< 0.01) and better subsequent communication and cooperation skills (Grade 1 best friend to Grade 2 cooperation *r *= 0.11, *p *< 0.01, and Grade 1 best friend to Grade 2 cooperation *r *= 0.13, *p *< 0.01). Having a best friend at age 4 was also associated with better cooperation skills in Grade 2 (*r *= 0.10, *p *< 0.01). Depression symptoms showed associations across time (age 4 and Grade 1: *r *= 0.19, *p *< 0.01; and Grades 1 and 2: *r *= 0.21, *p *< 0.01; age 4 and Grade 2: *r *= 0.19, *p *< 0.01) and were consistently negatively associated with cooperation and communication skills in Grade 1 (communication: *r*s = −0.28 to −0.33, *p *< 0.01; cooperation: *r*s =−0.23 to −0.28, *p *< 0.01) and Grade 2 (communication: *r*s =−0.25 to −0.38, *p *< 0.01; cooperation: *r*s =−0.18 to −0.31, *p *< 0.01). Communication and cooperation skills were associated within (Grade 1: *r *= 0.73, *p *< 0.01; Grade 2: *r *= 0.73, *p *< 0.01) and across time (Grade 1 to Grade 2 communication: *r *= 0.57, *p *< 0.01; Grade 1 to Grade 2 cooperation: *r *= 0.69, *p *< 0.01; Grade 1 communication to Grade 2 cooperation: *r *= 0.52, *p *< 0.01; Grade 1 cooperation and Grade 2 communication: *r *= 0.50, *p *< 0.01).

**Table 1:** Descriptive statistics.

	DEP age 4	DEP GR 1	DEP GR 2	COM GR 1	COM GR 2	COOP GR 1	COOP GR 2	Child gender	Early risk	PI age 4	Hours in daycare age 4	Mean	Standard deviation	Proportion (Yes)	*N*
Best friend age 4	**−0**.**120**	−0.030	−0.025	0.031	0.062	0.058	**0**.**101**		−0.030	−0.015	0.022			0.78	637
Best friend GR 1	−0.037	**−0**.**081**	−0.067	**0**.**130**	**0**.**108**	**0**.**109**	**0**.**129**		−0.037	−0.030	−0.043			0.56	871
Best friend GR 2	−0.044	−0.042	**−0**.**105**	0.053	**0**.**073**	0.053	**0**.**121**		**−0**.**089**	−0.040	0.008			0.60	897
DEP age 4		**0**.**205**	**0**.**192**	**−0**.**285**	**−0**.**249**	**−0**.**274**	**−0**.**184**	**−0**.**082**	**0**.**128**	**0**.**113**	−0.024	1.204	0.255		642
DEP GR 1			**0**.**577**	**−0**.**328**	**−0**.**276**	**−0**.**284**	**−0**.**245**	**−0**.**057**	**0**.**122**	**0**.**125**	0.017	1.127	0.218		873
DEP GR 2				**−0**.**284**	**−0**.**380**	**−0**.**233**	**−0**.**313**	**−0**.**115**	**0**.**202**	**0**.**131**	−0.070	1.128	0.215		898
COM GR 1					**0**.**574**	**0**.**726**	**0**.**518**	**0**.**229**	**−0**.**144**	**−0**.**098**	−0.069	22.919	3.236		863
COM GR 2						**0**.**503**	**0**.**727**	**0**.**252**	**−0**.**178**	**−0**.**094**	0.029	23.185	3.338		885
COOP GR 1							**0**.**690**	**0**.**288**	**−0**.**181**	**−0**.**08**	−0.026	19.671	3.231		868
COOP GR 2								**0**.**307**	**−0**.**219**	**−0**.**119**	0.015	19.591	3.379		886
Child gender									−0.022	0.024	0.015				942
Early risk										**0**.**266**	−0.014	0.419	0.737		916
PI age 4											0.080	1.285	0.355		932
Hours in daycare age 4												34.652	6.198		909

GR, Grade; DEP, depression symptoms; COM, communication; COOP, cooperation; PI, parental internalizing symptoms.

Best friend is a dichotomous variable and proportions are included for these variables. Bold represents correlations *p *< 0.05.

### 3.3 Cross-lagged panel model

We began with a model (Model 1) that included within time associations (covariances), auto-regressive stability paths among constructs across time, and cross-lagged associations among depression symptoms (age 4, Grade 1, Grade 2), having a best friend (age 4, Grade 1, Grade 2), communication skills (Grade 1, Grade 2), and cooperation skills (Grade 1, Grade 2) along with paths from early socioeconomic risk, hours in daycare at age 4, parental internalizing symptoms, and child gender to all variables in the model. Fit indices for the models conducted can be found in Table 2. Model 1 fit the data well [*χ^2^*(16) = 26.404, *p *= 0.049; *CFI *= 0.995; *RMSEA *= 0.026, 95% CI 0.002–0.044].

**Table 2:** Fit indices for cross-lagged panel model of depression symptoms, cooperation, communication skills, and best friend.

Model	*CFI*	*RMSEA* (90% CI)	*χ* ^2^	df	*p*	Comparison	χ^2^	df	*p*-value
1. Overall model including auto-regressive, covariance, and cross-lagged paths among depression, cooperation, communication and best friend with covariates of early risk, gender of child, parental mental health, and hours in daycare	0.995	.0026 (0.002–0.044)	26.404	16	0.049				
2. Final model with gender of child, early risk, and parental internalizing symptoms; constraints incorporated	0.996	0.019 (0.000–0.034)	32.043	24	0.126	2 vs. 1	6.044	8	0.642

Next, from Model 1, each set of paths that could be constrained to be equal across time were separately constrained to estimate the most parsimonious model similar to procedures in previous research (48). We found that constraining paths from depression symptoms to best friend [*χ^2^*(1) = 0.014, *p *= 0.91], best friend to depression symptoms [*χ^2^*(1) = 0.069, *p *= 0.79], best friend to communication [*χ^2^*(1) = 0.013, *p *= 0.91], best friend to cooperation [*χ^2^*(1) = 0.169, *p *= 0.68], and within time associations between depression symptoms and best friends [*χ^2^*(2) = 2.313, *p *= 0.315], communication and best friend [*χ^2^*(1) = 2.07, *p *= 0.15], and cooperation and best friend [*χ^2^*(1) = 0.043, *p *= 0.84] did not result in a statistically significant change in model fit so these constraints were retained in the final model. We found that constraining similar paths of depression symptoms to cooperation [age 4 to Grade 1, and Grade 1 to Grade 2: *χ^2^*(1) = 13.357, *p *< 0.01], depression symptoms to communication [age 4 to Grade 1, and Grade 1 to Grade 2: *χ^2^*(1) = 14.978, *p *< 0.01], stability of best friend [age 4 to Grade 1, and Grade 1 to Grade 2: *χ^2^*(1) = 13.73, *p *< 0.01], and within time associations between cooperation and communication [*χ^2^*(1) = 21.084, *p *< 0.01], depression symptoms and cooperation [*χ^2^*(1) = 7.301, *p *< 0.01], depression symptoms and communication [*χ^2^*(1) = 9.531, *p *< 0.01] degraded model fit and so were freely estimated in the final model. Constraining the auto-regressive paths for depression symptoms (age 4 to Grade 1, and Grade 1 to Grade 2) did not converge so these were freely estimated in the final model.

**Figure 1:**
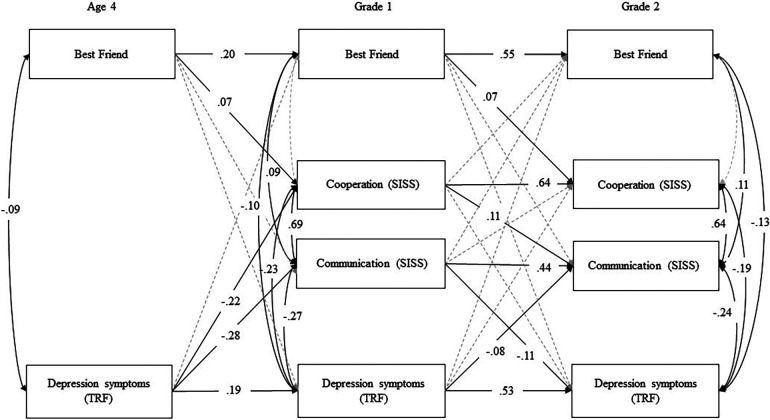
Cross-lagged panel model of depression symptoms, cooperation, communication, and best friend with covariates of gender of child, early risk, and parental internalizing symptoms paths (model 2: final model with constraints incorporated). Note: Standardized estimates. See Table 2 for fit indices and Supplementary Table S3 for all unstandardized and standardized path estimates. Gray paths were estimated but were not statistically significant.

A final model (Model 2) was conducted including the associations in Model 1 and the constraints from depression to best friend, best friend to depression, best friend to communication, best friend to cooperation, depression symptoms and best friends, communication and best friend, and cooperation and best friend were retained. The remaining paths that were repeated over time and showed degradation in model fit with the constraints were allowed to be freely estimated. This model also fit the data well [*χ^2^*(24) = 32.043, *p *= 0.56; *CFI *= 0.996; *RMSEA *= 0.019, 95% CI 0.000−0.034], did not statistically significantly differ from Model 1 [*χ^2^*(8) = 6.044, *p *= 0.642], and is depicted in Figure 1.

All unstandardized and standardized coefficients for Model 2 are provided in Supplementary Table S3. In this model, we found that having a best friend showed stability (i.e., age 4 predicted Grade 1: β = 0.20, *p *< 0.01; Grade 1 predicted Grade 2: β = 0.55, *p *< 0.01), depression symptoms showed stability (i.e., age 4 predicted Grade 1: β = 0.19, *p *< 0.01; Grade 1 predicted Grade 2: β = 0.53, *p *< 0.01), and communication skills (β = 0.44, *p *< 0.01) and cooperation skills (β = 0.64, *p *< 0.01) showed stability from Grade 1 to Grade 2. At age 4 (*r *= −0.09, *p *< 0.01), Grade 1 (*r *=−0.10, *p *< 0.01), and in Grade 2 (*r *= −0.13, *p *< 0.01), not having a best friend was concurrently associated with depression symptoms. Having a best friend predicted later cooperation skills (i.e., age 4 to Grade 1: β = 0.07, *p *= 0.033; Grade 1 to Grade 2: β = 0.07, *p *= 0.033). In Grade 1, having a best friend was positively associated with communication skills (*r *= 0.09, *p *< 0.01) and in Grade 2 (*r *= 0.11, *p *< 0.01). Depression symptoms were negatively associated with communication (Grade 1: *r *= −0.27, *p *< 0.01; Grade 2: *r *= −0.24, *p *< 0.01) and cooperation skills (Grade 1: *r *= −0.23, *p *< 0.01; Grade 2: *r *= −0.19, *p *< 0.01) within time at both Grade 1 and Grade 2. Communication and cooperation skills were also positively associated at both time points (Grade 1: *r *= 0.69, *p *< 0.01; Grade 2: *r *= 0.64, *p *< 0.01). Depression symptoms at age 4 were negatively associated with communication (β = −0.28, *p *< 0.01) and cooperation (β = −0.22, *p *< 0.01) skills in Grade 1. Depression symptoms in Grade 1 were also significantly negatively associated with communication skills in Grade 2 (β = −0.08, *p *< 0.01), but not with Grade 2 cooperation skills (β = −0.04, *p *= 0.14). Communication skills in Grade 1 were negatively related to depression symptoms in Grade 2 (β = −0.11, *p *< 0.01), and cooperation skills in Grade 1 positively predicted communication skills in Grade 2 (β = 0.11, *p *< 0.01).

#### 3.3.1 Indirect effects

**Figure 2:**
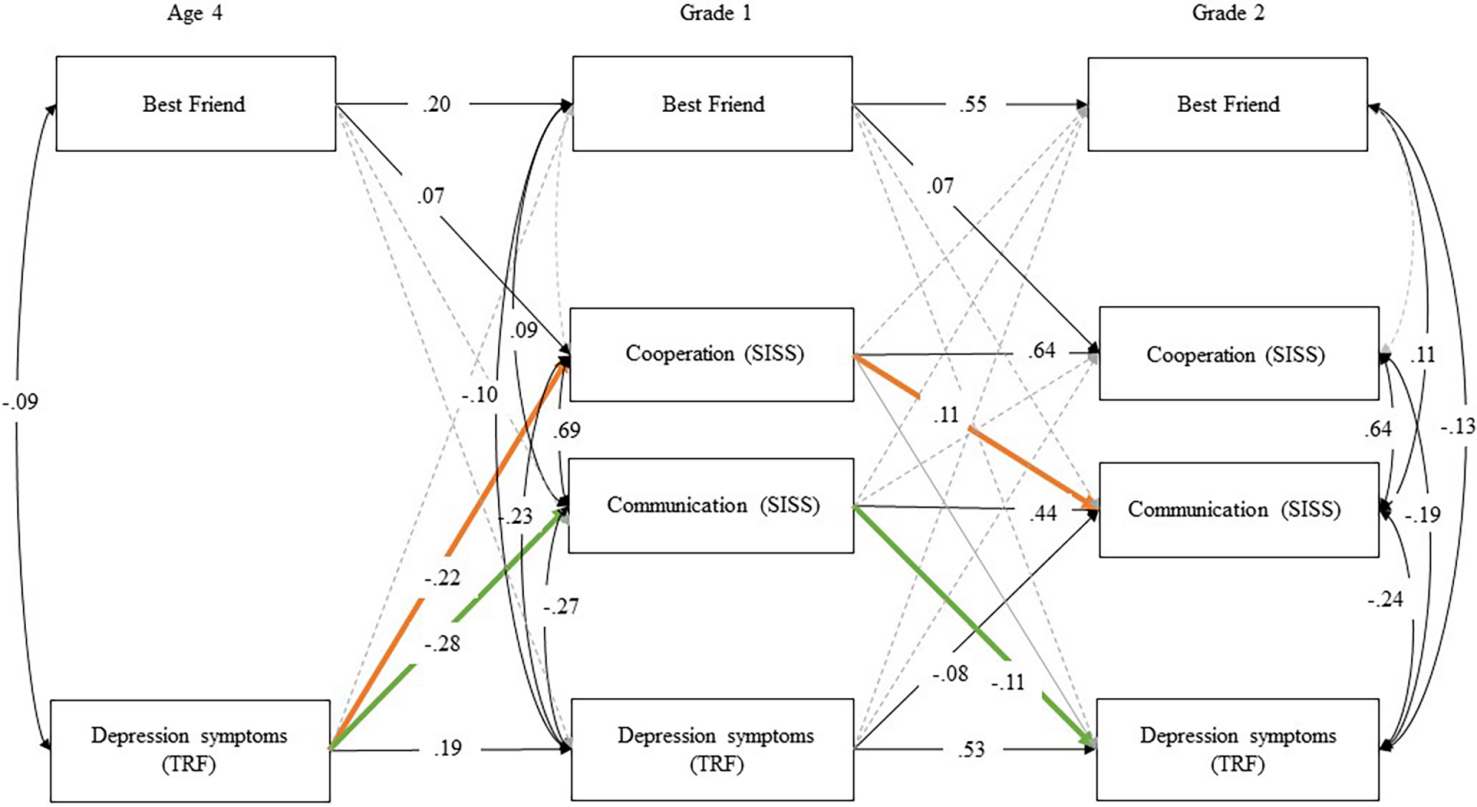
Indirect effects of cross-lagged panel model of depression symptoms, cooperation, communication, and best friend with covariates of gender of child, early risk, and parental internalizing symptoms paths (model 2: final model with constraints incorporated.). Note: Standardized estimates. See Table 2 for fit indices and Supplementary Table S3 for all unstandardized and standardized path estimates. Gray paths were estimated but were not statistically significant. Indirect effects: Age 4 depression symptoms (TRF) → Grade 1 communication (SSIS) → Grade 2 depression symptoms (TRF) *b *= 0.026, β = 0.031, 95% CI (0.009, 0.043); Age 4 depression symptoms (TRF) → Grade 1 cooperation (SSIS) → Grade 2 communication (SSIS) *b * =−0.312, β =− 0.024, 95% CI (−0.549, −0.076).

Two indirect effects were tested: (1) age 4 depression symptoms to Grade 1 communication skills to Grade 2 depression symptoms [*b *= 0.026, β = 0.031, 95% CI (0.009, 0.043)], (2) age 4 depression symptoms to Grade 1 cooperation skills to Grade 2 communication skills [*b *= −0.312, β = −0.024, 95% CI (−0.549, −0.076)] and both were statistically significant as depicted in Figure 2.

#### 3.3.2 Covariates

The covariates also had multiple statistically significant associations within the model. The path estimates (unstandardized and standardized) related to the covariates can be observed in Table 3. Specifically, early socioeconomic risk was positively related to depression symptoms at every time point with the exceptions of having a best friend at age 4 and in Grade 1, negatively related to communication and cooperation skills at both time points, and related to not having a best friend in Grade 2. Parental internalizing symptoms when the child was 4 years old predicted depression symptoms at age 4 and Grade 1 but not at Grade 2, and negatively predicted Grade 2 cooperation skills. Parental internalizing symptoms and early socioeconomic risk were also significantly associated. Being a boy was associated with more depression symptoms at age 4 and in Grade 2. Being a girl was associated with better communication and cooperation skills at both time points and having a best friend in Grade 1.

## 4 Discussion

Our aim was to assess the longitudinal associations between depression symptoms in early childhood, having a best friend, and early school-age social skills of cooperation and communication. We found that young children experiencing depression symptoms were less likely to have a best friend (age 4, Grade 1, and Grade 2) and their communication and cooperation skills were concurrently impaired. Impairment in communication and cooperation skills also persisted over time.

The results suggested an indirect effect where depression symptoms at age 4 negatively predicted communication skills in Grade 1, which in turn negatively predicted depression symptoms in Grade 2. Those with higher depression symptoms at age 4 had poorer communication skills in Grade 1, which was associated with higher depression symptoms in Grade 2. This may not be a surprising finding since depressed children are shown to interact less with others (3) and are thus likely to have fewer opportunities to practice their communication skills with their peers. That is, a higher frequency of negative peer interactions and fewer opportunities to interact likely interferes with the reinforcement needed to effectively establish good communication skills. Since communication skills are important for developing friendships (30), impairment in this area may have an impact on peer relations. Given the protective role of friendship for young children with genetic predispositions for depression symptoms (10), support in developing communication skills may help children build friendships. Still, to maintain such friendships, cooperation skills may also be necessary (30).

Depression symptoms at age 4 negatively predicted cooperation in Grade 1, but was not consistent over time (i.e., the path from depressive symptoms in Grade 1 to cooperation skills in Grade 2 was not statistically significant). Cooperation skills in Grade 1 positively predicted communication skills in Grade 2. The indirect effect from age 4 depression symptoms to Grade 1 cooperation to Grade 2 communication skills was significant, meaning that higher depression symptoms at age 4 were related to lower cooperation skills in Grade 1, which in turn were related to lower communication skills in Grade 2. Early depression symptoms may be leading to deficits in cooperation and communication skills over time, which are important for building and maintaining friendships (30). Since depression symptoms are reported both within the preschool/daycare context by daycare teachers and the formal school setting by teachers, the presence of these indirect effects from preschool into Grade 2 are noteworthy and require replication.

Higher depression symptoms at age 4 were associated with not having a best friend at each time point.

The positive associations between having a best friend and later cooperation skills were present from age 4 to Grade 1 and Grade 1 to Grade 2. This supports the literature suggesting cooperation skills are important in the maintenance of friendships in early childhood and our model suggests that having a best friend predicts later cooperation skills and not the reverse (30). Early relationships are likely important for the development of cooperation skills in young children. Conversely, when these relationships are not present in the lives of young children, more support in developing cooperation skills should be considered for future friendship maintenance.

Being a girl was associated with better communication and cooperation skills in Grades 1 and 2. Better cooperation skills have been found for girls in previous research (32). Girls tend to acquire language skills more quickly than boys and developmental disorders involving communication are more prevalent in boys (49). This may relate to the earlier onset of language within girls, which impacts communication and cooperation skills (50). There is established research literature supporting that communication and language abilities advance more quickly for girls and this finding is robust (51).

Early socioeconomic risk was associated with not having a best friend in Grade 2, higher depression symptoms at each time point, and lower cooperation and communication skills across time. Socially disadvantaged children have been found to experience language delays, including general communication skills, also in relatively affluent societies such as the Norwegian (52, 53). Parental self-reported internalizing symptoms were associated with teacher-rated depression symptoms at age 4 and in Grade 1 and were correlated with early socioeconomic risk. Being a boy was associated with depression symptoms at age 4 and in Grade 2. The gender difference in depression symptoms favoring girls occurs after puberty; before puberty, the rates of depression are low in both girls and boys (54). Due to the low frequency of depression symptoms in early childhood in the general population and the tendency for studies in early childhood to have smaller sample sizes, the inconsistency in gender differences in early childhood may relate to lack of power to detect the effects. More research is needed with larger samples examining gender differences in depression symptoms to better understand the development for boys and girls.

The early socioeconomic risk measured at 6 and 12 months was associated with most study variables. There is a long history of socioeconomic status conferring risk for poor mental health among children, particularly for indicators related to low income and parental education (55–57). Either parent having internalizing symptoms was a risk factor for concurrent and later depression symptoms in children, consistent with the well-established literature linking parental psychopathology and child maladjustment (58, 59). A previous study shows that children in Grade 2 make significant gains in social skill development of communication and cooperation, and this corresponded with a decrease in internalizing symptoms (20). However, in another study there was no statistically significant change in internalizing symptoms in Grade 1 and the effect sizes of gains in social skills were half of those found in the (20) study (60). It may be that a certain amount of skill development needs to occur before this has an influence on internalizing symptoms. The impact of interventions for cooperation and communication skills and depression symptoms in young children, particularly those experiencing early socioeconomic risk, needs to be evaluated in future research.

Our study had significant strengths, including a relatively large sample for early childhood studies that was longitudinal with little attrition (33) and good measurement of constructs. However, as is the case with all studies, there are limitations. First, a composite of consistent items of child depression symptoms could not be created from age 4 to Grade 1 and Grade 2. The older children had consistent items from Grade 1 to Grade 2 and therefore did not have this issue. Future research should use consistent items over all time points to replicate our findings. Second, we did not have corresponding measures of cooperation and communication skills at age 4, thus it is possible that deficits in these skills may have started earlier than Grade 1. Third, we measured best friends at school and children could have had a best friend in a different context (e.g., neighborhood, family, extracurricular activities). Fourth, there are likely other covariates that could have an impact on our results. For example, depression symptoms have been linked to academic performance (61). Finally, we examined child gender as a covariate instead of in a multi-group model due to problems with convergence. Studies with larger samples should examine how gender moderates the associations between depression symptoms, communication, and cooperation skills, and having a best friend in young children over time.

### 4.1 Implications

Our results suggest that early intervention for depression symptoms should be considered in relation to social skill development in the early school-age years. Programs aimed at the prevention of internalizing symptoms in the preschool period do exist and have some evidence of efficacy in the short term (62) and into middle childhood for girls (63). Further, some children, particularly those with elevated depression symptoms, may also benefit from more support in developing cooperation and communication skills. Our indirect effect of depression symptoms predicting communication deficits, which in turn predicts depression symptoms, suggests that poor communication skill development may be a maintenance factor for depression symptoms in young children. Importantly, communication skills are modifiable and one of the social skills that is more consistently influenced by intervention efforts (20). More research is needed to determine whether, or at what point during or after treatment of depression symptoms, communication skills training influences the development of children's friendships. There is some support for social-emotional learning influencing emotional distress for school-aged children (kindergarten to Grade 12) (64). Participating in social skills training programs has also shown to somewhat decrease internalizing symptoms, with the largest effect sizes for the skills of cooperation and communication (20). A meta-analysis showed that class-wide social skills interventions in preschool, elementary, and secondary school were minimally effective; however, younger children participating in the program (i.e., preschool and kindergarten) seemed to have benefited the most from the program (65). A targeted approach for those who are more at risk of social skill deficits may also play an important role in supporting the equitable development of social skills. In line with this, those who experience heightened early socioeconomic risk, increased parental internalizing symptoms (of either parent), and being boys may be groups that warrant intervention and prevention efforts for depression symptoms, communication skills, and cooperation skills. The cost of implementing these programs is small in comparison to the cost of treatment once diagnostic criteria are met; therefore, the public health benefit in the long term may be significant with investment in early childhood mental health programs (63).

In sum, we showed prospective associations between symptoms of depression and poorer social skills (cooperation, communication, and friendships) in early childhood and the first few years of school. The temporal pattern of findings suggests that deficits in communication skills may be a maintenance factor for depression symptoms and that deficits in cooperation skills further degrade communication skills. Early socioeconomic risk, parental internalizing symptoms, and child gender, were associated with depression symptoms and cooperation and communication skills. Given that depression symptoms negatively impact the development of communication skills, cooperation skills, and friendships, it is important to intervene early with young children who have depression symptoms.

## Data Availability

The data analyzed in this study are subject to the following licenses/restrictions: The data are part of an ongoing longitudinal study and contain potentially sensitive information. The consent of the participants of the Behavior Outlook Norwegian Developmental Study (BONDS), as approved by the Regional Committee for Medical and Health Research Ethics in South-East Norway does not include sharing a de-identified data set on an open server. In compliance with the approval and consent for the study, researchers who meet the criteria for access to confidential data may be given access to de-identified data via a secure server, under a data processing agreement. Requests for data access should be directed to the Norwegian Center for Child Behavioral Development (NUBU), Oslo, Norway (mail: post@nubu.no) or to the corresponding author (ane.narde@nubu.no).
