# Supplementary material for: Depression symptoms, communication and cooperation skills, and friendship: longitudinal associations in young Norwegian children
Source: Front Child Adolesc Psychiatry. 2024 Aug 22;3:1328527. doi: 10.3389/frcha.2024.1328527 (PMC11731607; doi:10.3389/frcha.2024.1328527)
Supplement: Supplementary file 1 [file Table1.docx]

Supplementary Material

# Supplementary Table

Table 3. Unstandardized and standardized path estimates for final cross-lagged panel model.

|  | *b* | *p* | *B* | *p* |
| --- | --- | --- | --- | --- |
| BF age 4 to BF Grade 1 | 0.204 | 0.009 | 0.197 | 0.006 |
| DEP Age 4 to BF Grade 1 | -0.019 | 0.899 | -0.005 | 0.912 |
| early risk to BF Grade 1 | -0.027 | 0.676 | -0.020 | 0.676 |
| PI age 4 to BF Grade 1 | -0.125 | 0.342 | -0.043 | 0.341 |
| gender to BF Grade 1 | 0.342 | 0.000 | 0.165 | 0.000 |
| BF Grade 1 to BF Grade 2 | 0.642 | 0.000 | 0.552 | 0.000 |
| COOP Grade 1 to BF Grade 2 | 0.011 | 0.647 | 0.029 | 0.646 |
| COM to BF Grade 2 | -0.023 | 0.333 | -0.061 | 0.313 |
| DEP Age 4 to BF Grade 2 | 0.019 | 0.912 | 0.003 | 0.912 |
| early risk to BF Grade 2 | -0.147 | 0.044 | -0.091 | 0.043 |
| PI age 4 to BF Grade 2 | -0.014 | 0.918 | -0.004 | 0.918 |
| gender to BF Grade 2 | -0.068 | 0.521 | -0.028 | 0.520 |
| DEP age 4 to DEP Grade 1 | 0.163 | 0.000 | 0.192 | 0.000 |
| BF age 4 to DEP Grade 1 | 0.000 | 0.949 | 0.002 | 0.949 |
| early risk to DEP Grade 1 | 0.019 | 0.048 | 0.066 | 0.047 |
| PI age 4 to DEP Grade 1 | 0.051 | 0.007 | 0.083 | 0.007 |
| gender to DEP Grade 1 | -0.017 | 0.257 | -0.038 | 0.257 |
| DEP Grade 1 to DEP Grade 2 | 0.524 | 0.000 | 0.528 | 0.000 |
| BF Grade 1 to DEP Grade 2 | 0.000 | 0.949 | 0.002 | 0.949 |
| COOP Grade 1 to DEP Grade 2 | 0.003 | 0.150 | 0.05 | 0.151 |
| COM Grade 1 to DEP Grade 2 | -0.007 | 0.001 | -0.109 | 0.001 |
| early risk to DEP Grade 2 | 0.039 | 0.000 | 0.135 | 0.000 |
| PI to DEP Grade 2 | 0.017 | 0.211 | 0.029 | 0.210 |
| gender to DEP Grade 2 | -0.031 | 0.020 | -0.072 | 0.019 |
| COOP Grade 1 to COOP Grade 2 | 0.666 | 0.000 | 0.638 | 0.000 |
| BF Grade 1 to COOP Grade 2 | 0.226 | 0.030 | 0.069 | 0.030 |
| COM Grade 1 to COOP Grade 2 | -0.025 | 0.528 | -0.024 | 0.528 |
| DEP Grade 1 to COOP Grade 2 | -0.587 | 0.139 | -0.038 | 0.138 |
| early risk to COOP Grade 2 | -0.423 | 0.000 | -0.093 | 0.000 |
| PI age 4 to COOP Grade 2 | -0.391 | 0.066 | -0.041 | 0.065 |
| gender to COOP Grade 2 | 0.771 | 0.000 | 0.114 | 0.000 |
| COM Grade 1 to COM Grade 2 | 0.456 | 0.000 | 0.441 | 0.000 |
| BF Grade 1 to COM Grade 2 | 0.060 | 0.563 | 0.019 | 0.564 |
| COOP Grade 1 to COM Grade 2 | 0.112 | 0.003 | 0.109 | 0.003 |
| DEP Grade 1 to COM Grade 2 | -1.199 | 0.000 | -0.078 | 0.000 |
| early risk to COM Grade 2 | -0.403 | 0.001 | -0.090 | 0.001 |
| PI age 4 to COM Grade 2 | -0.085 | 0.762 | -0.009 | 0.762 |
| gender to COM Grade 2 | 0.744 | 0.000 | 0.111 | 0.000 |
| BF age 4 to COOP Grade 1 | 0.226 | 0.030 | 0.070 | 0.031 |
| DEP age 4 to COOP Grade 1 | -2.792 | 0.000 | -0.220 | 0.000 |
| early risk to COOP Grade 1 | -0.615 | 0.000 | -0.142 | 0.000 |
| PI to COOP Grade 1 | -0.255 | 0.377 | -0.028 | 0.338 |
| gender to COOP Grade 1 | 1.704 | 0.000 | 0.263 | 0.000 |
| BF age 4 to COM Grade 1 | 0.060 | 0.563 | 0.019 | 0.564 |
| DEP age 4 to COM Grade 1 | -3.548 | 0.000 | -0.280 | 0.000 |
| early risk to COM Grade 1 | -0.391 | 0.001 | -0.090 | 0.001 |
| PI to COM Grade 1 | -0.509 | 0.041 | -0.05 | 0.065 |
| gender to COM Grade 1 | 1.321 | 0.000 | 0.205 | 0.000 |
| early risk to BF age 4 | -0.112 | 0.192 | -0.083 | 0.190 |
| Daycare hours age 4 to BF age 4 | 0.001 | 0.911 | 0.06 | 0.911 |
| PI to BF age 4 | 0.092 | 0.560 | 0.033 | 0.559 |
| gender to BF age 4 | 0.011 | .928 | 0.005 | 0.928 |
| early risk to DEP age 4 | -0.043 | 0.022 | -0.088 | 0.021 |
| Daycare hours age 4 to DEP age 4 | -0.001 | 0.73 | -0.013 | 0.730 |
| PI to DEP age 4 | 0.052 | 0.016 | 0.073 | 0.016 |
| gender to DEP age 4 | -0.045 | 0.022 | -0.088 | 0.021 |
| BF age 4 with DEP age 4 | -0.021 | 0.001 | -0.086 | 0.001 |
| DEP Grade 1 with BF Grade 1 | -0.021 | 0.001 | -0.102 | 0.001 |
| COM Grade 1 with BF Grade 1 | 0.275 | 0.008 | 0.093 | 0.008 |
| COOP Grade 1 with BF Grade 1 | 0.186 | 0.100 | 0.063 | 0.098 |
| DEP Grade 1 with COM Grade 1 | -0.168 | 0.000 | -0.269 | 0.000 |
| DEP Grade 1 with COOP Grade 1 | -0.142 | 0.000 | -0.229 | 0.000 |
| COM Grade 1 with COOP Grade 1 | 6.009 | 0.000 | 0.689 | 0.000 |
| DEP Grade 2 with BF Grade 2 | -0.021 | 0.001 | -0.125 | 0.001 |
| COM Grade 2 with BF Grade 2 | 0.275 | 0.008 | 0.105 | 0.009 |
| COOP Grade 2 with BF Grade 2 | 0.186 | 0.100 | 0.079 | 0.068 |
| DEP Grade 2 with COM Grade 2 | -0.106 | 0.000 | -0.237 | 0.000 |
| DEP Grade 2 with COOP Grade 2 | -0.760 | 0.000 | -0.188 | 0.000 |
| COM Grade 2 with COOP Grade 2 | 3.920 | 0.000 | 0.636 | 0.000 |
| gender with early risk | -0.006 | 0.634 | -0.016 | 0.634 |
| gender with daycare hours age 4 | 0.013 | 0.909 | 0.004 | 0.909 |
| gender with PI age 4 | 0.004 | 0.453 | 0.025 | 0.467 |
| PI age 4 with early risk | 0.072 | 0.000 | 0.271 | 0.000 |
| PI age 4 with daycare hours age 4 | 0.176 | 0.019 | 0.080 | 0.018 |
| Daycare hours age 4 with early risk | -0.097 | 0.538 | -0.021 | 0.538 |
| Note. DEP=depression symptoms; COM=communication; COOP=cooperation; PI=parental internalizing symptoms; BF=best friend. 92 parameters in the model. | | | | |
